# A triclinic polymorph of methyl (3*R*,3′*S*)-1′,1′′-dimethyl-2,2′′-dioxodispiro­[indoline-3,2′-pyrrolidine-3′,3′′-indoline]-4′-carboxyl­ate

**DOI:** 10.1107/S160053681204706X

**Published:** 2012-11-28

**Authors:** G. Ganesh, Panneer Selvam Yuvaraj, Chinthalapuri Divakara, Boreddy S. R. Reddy, A. SubbiahPandi

**Affiliations:** a Department of Physics, S.M.K. Fomra Institute of Technology, Thaiyur, Chennai 603 103, India; b Industrial Chemistry Laboratory, Central Leather Research Institute, Adyar, Chennai 600 020, India; c Department of Physics, Presidency College (Autonomous), Chennai 600 005, India

## Abstract

In the title compound, C_22_H_21_N_3_O_4_, the central pyrrolidine ring adopts a C-envelope conformation with a C atom 0.6593 (13) Å displaced from the mean plane formed by the remaining ring atoms. The indoline ring systems (r.m.s. devisations of 0.0356 and 0.0547 Å) are almost perpendicular to the mean plane of the pyrrolidine ring, making dihedral angles of 89.7 (6) and 82.5 (6)°. The acetate group attached to the pyrrolidine ring assumes an extended conformation. In the crystal,N—H⋯O and C—H⋯O hydrogen bonds connect adjacent molecules, forming an infinite tape extending along [1-1-1]. The crystal packing is further consolidated by strong π–π inter­actions with a centroid–centroid distance of 3.2585 (8) Å. The title compound is a polymorph of previously reported monoclinic structure [Ganesh *et al.* (2012 ▶). *Acta Cryst.* E**68**, o2902–o2903].

## Related literature

For background literature and the previously reported polymorph, see: Ganesh *et al.* (2008). For a related structure, see: Wei *et al.* (2011 ▶).
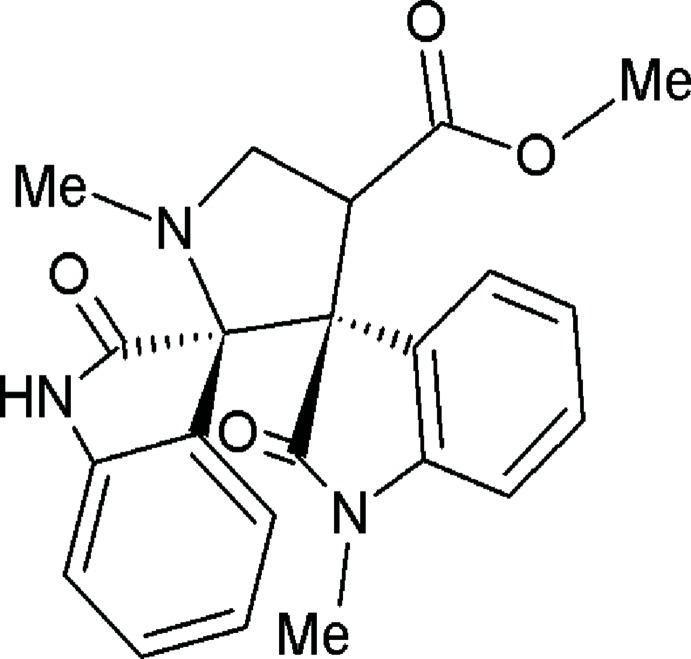


## Experimental

### 

#### Crystal data

C_22_H_21_N_3_O_4_
*M* *_r_* = 391.42
Triclinic, 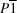

*a* = 9.4418 (3) Å
*b* = 10.0132 (3) Å
*c* = 12.8861 (4) Å
α = 67.465 (2)°
β = 88.237 (2)°
γ = 62.842 (1)°
*V* = 985.68 (5) Å^3^
*Z* = 2
Mo *K*α radiation
μ = 0.09 mm^−1^
*T* = 293 K
0.25 × 0.22 × 0.19 mm

#### Data collection

Bruker APEXII CCD area-detector diffractometer
Absorption correction: multi-scan (*SADABS*; Bruker, 2008 ▶) *T*_min_ = 0.978, *T*_max_ = 0.983
23829 measured reflections
8469 independent reflections
5108 reflections with *I* > 2σ(*I*)
*R*_int_ = 0.028

#### Refinement

*R*[*F* ^2^ > 2σ(*F* ^2^)] = 0.051
*wR*(*F* ^2^) = 0.158
*S* = 1.08
8469 reflections
265 parameters
H-atom parameters constrained
Δρ_max_ = 0.29 e Å^−3^
Δρ_min_ = −0.27 e Å^−3^

### 

Data collection: *APEX2* (Bruker, 2008 ▶); cell refinement: *SAINT* (Bruker, 2008 ▶); data reduction: *SAINT*; program(s) used to solve structure: *SHELXS97* (Sheldrick, 2008 ▶); program(s) used to refine structure: *SHELXL97* (Sheldrick, 2008 ▶); molecular graphics: *ORTEP-3* (Farrugia, 2012) ▶; software used to prepare material for publication: *SHELXL97* and *PLATON* (Spek, 2009 ▶).

## Supplementary Material

Crystal structure: contains datablock(s) global, I. DOI: 10.1107/S160053681204706X/pv2603sup1.cif

Structure factors: contains datablock(s) I. DOI: 10.1107/S160053681204706X/pv2603Isup2.hkl

Additional supplementary materials:  crystallographic information; 3D view; checkCIF report

## Figures and Tables

**Table 1:** Hydrogen-bond geometry (Å, °)

*D*—H⋯*A*	*D*—H	H⋯*A*	*D*⋯*A*	*D*—H⋯*A*
N3—H3⋯O1^i^	0.86	2.10	2.9107 (12)	157
C15—H15*B*⋯O1^ii^	0.96	2.47	3.363 (2)	155

Symmetry codes: (i) 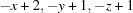; (ii) 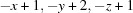.

**Fig. 1.**
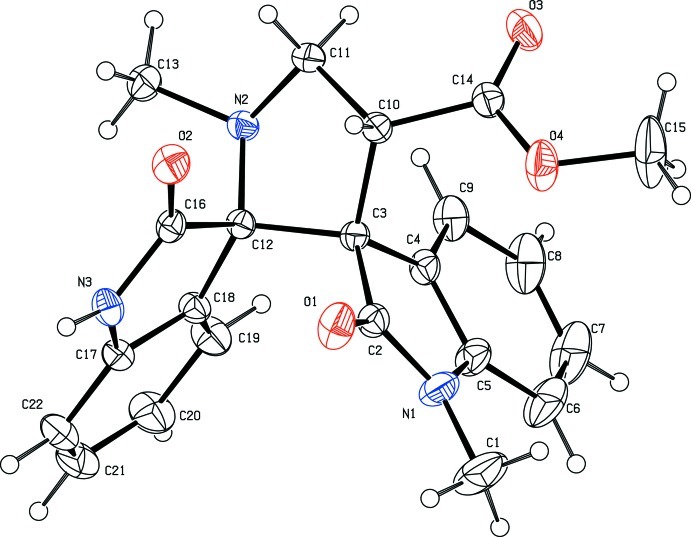
The molecular structure of the title compound with the atom numbering scheme. Displacement ellipsoids are drawn at the 30% probability level. H atoms are presented as small spheres of arbitrary radius.

**Fig. 2.**
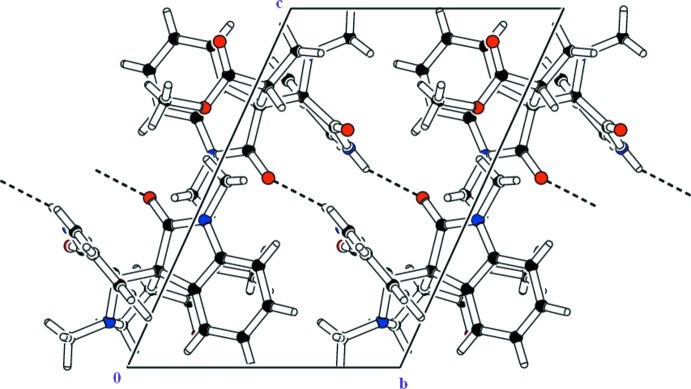
A view of the hydrogen bonds (dotted lines) in the crystal structure of the title compound. H atoms non-participating in hydrogen- bonding were omitted for clarity.

## supplementary crystallographic information

### Comment

We have recently reported the structure of the title compound in the monoclinic system (Ganesh *et al.*, 2012). Here we report the structural details of its triclinic polymorph.

The bond lengths and angles in the title molecule (Fig. 1) are within normal ranges and comparable to those reported for its triclinic polymorph (Ganesh *et al.*, 2012) and a closely related structure (Wei *et al.*, 2011). The indoline ring systems (N1/C2-C9 and N3/C12/C16-C22) are individually planar (rmsd's 0.0356 and 0.0547 Å, respectively) and make dihedral angles of 89.69 (6) ° and 82.48 (6)° with respect to the mean plane of the pyrrolidine ring system (N2/C3/C10–C12). The pyrrolidine ring [N2/C3/C10-C12] adopts a C12-envelop conformation with C12 0.6593 (13) Å displaced from the mean-plane formed by the remaining ring atoms. The acetate group assumes an extended conformation (torsion angle C10–C14–O4–C15 = 176.3 (2) °).

The crystal structure is stabilized by intermolecular N3—H3···O1 and C15—H15B···O1 hydrogen bonds. There are strong π–π interactions with a centroid-centroid distance of 3.2585 (8) Å between *Cg*1 and *Cg*3 rings. {*Cg*1 and *Cg*3 are the centroids of the N1/C2-C5 and N3/C12/C16-C18 rings respectively}.

### Experimental

A mixture of 1 eq of (*E*)-methyl 2-(1-methyl-2-oxoindolin-3-ylidene) acetate, 1 eq of isatin and 1.5 eq of sarcosine dissolved in acetonitrile was refluxed at 353 K for 8 h. Upon completion of the reaction as determined with the aid of TLC, the reaction mixture was extracted with ethyl acetate and water. The product was dried and purified by column chromatography using ethyl acetate and hexane (1:9) as an elutent to afford the title compound in pure form. (Yield = 90%). Single crystals suitable for X-ray diffraction were obtained by slow evaporation of a solution of the title compound in ethyl acetate at room temperature.

### Refinement

All H atoms were fixed geometrically and allowed to ride on their parent C atoms, with N—H = 0.86 Å and C—H distances in the range 0.93–0.98 Å with *U*_iso_(H) = 1.5*U*_eq_(methyl C) and 1.2*U*_eq_(non-methyl C/N).

### Crystal data

**Table:**

C_22_H_21_N_3_O_4_	*Z* = 2
*M**_r_* = 391.42	*F*(000) = 412
Triclinic, *P*1	*D*_x_ = 1.319 Mg m^−^^3^
Hall symbol: -P 1	Mo *K*α radiation, λ = 0.71073 Å
*a* = 9.4418 (3) Å	Cell parameters from 8469 reflections
*b* = 10.0132 (3) Å	θ = 2.4–34.7°
*c* = 12.8861 (4) Å	µ = 0.09 mm^−^^1^
α = 67.465 (2)°	*T* = 293 K
β = 88.237 (2)°	Block, colourless
γ = 62.842 (1)°	0.25 × 0.22 × 0.19 mm
*V* = 985.68 (5) Å^3^	

### Data collection

**Table:**

Bruker APEXII CCD area-detector diffractometer	8469 independent reflections
Radiation source: fine-focus sealed tube	5108 reflections with *I* > 2σ(*I*)
Graphite monochromator	*R*_int_ = 0.028
ω and φ scans	θ_max_ = 34.7°, θ_min_ = 2.4°
Absorption correction: multi-scan (*SADABS*; Bruker, 2008)	*h* = −15→15
*T*_min_ = 0.978, *T*_max_ = 0.983	*k* = −16→16
23829 measured reflections	*l* = −20→20

### Refinement

**Table:**

Refinement on *F*^2^	Primary atom site location: structure-invariant direct methods
Least-squares matrix: full	Secondary atom site location: difference Fourier map
*R*[*F*^2^ > 2σ(*F*^2^)] = 0.051	Hydrogen site location: inferred from neighbouring sites
*wR*(*F*^2^) = 0.158	H-atom parameters constrained
*S* = 1.08	*w* = 1/[σ^2^(*F*_o_^2^) + (0.0823*P*)^2^ + 0.0657*P*] where *P* = (*F*_o_^2^ + 2*F*_c_^2^)/3
8469 reflections	(Δ/σ)_max_ < 0.001
265 parameters	Δρ_max_ = 0.29 e Å^−^^3^
0 restraints	Δρ_min_ = −0.27 e Å^−^^3^

### Special details

**Table:**

Geometry. All esds (except the esd in the dihedral angle between two l.s. planes) are estimated using the full covariance matrix. The cell esds are taken into account individually in the estimation of esds in distances, angles and torsion angles; correlations between esds in cell parameters are only used when they are defined by crystal symmetry. An approximate (isotropic) treatment of cell esds is used for estimating esds involving l.s. planes.
Refinement. Refinement of F^2^ against ALL reflections. The weighted R-factor wR and goodness of fit S are based on F^2^, conventional R-factors R are based on F, with F set to zero for negative F^2^. The threshold expression of F^2^ > 2sigma(F^2^) is used only for calculating R-factors(gt) etc. and is not relevant to the choice of reflections for refinement. R-factors based on F^2^ are statistically about twice as large as those based on F, and R- factors based on ALL data will be even larger.

### Fractional atomic coordinates and isotropic or equivalent isotropic displacement parameters (Å^2^)

**Table:**

	*x*	*y*	*z*	*U*_iso_*/*U*_eq_	
C1	0.7776 (3)	1.0526 (3)	0.51871 (15)	0.0796 (6)	
H1A	0.6672	1.1089	0.5272	0.119*	
H1B	0.8311	1.1134	0.5210	0.119*	
H1C	0.8314	0.9442	0.5797	0.119*	
C2	0.77232 (13)	0.91697 (13)	0.39598 (9)	0.0340 (2)	
C3	0.76315 (11)	0.95234 (11)	0.26878 (8)	0.02661 (19)	
C4	0.76056 (12)	1.11630 (12)	0.21751 (10)	0.0322 (2)	
C5	0.77058 (15)	1.16217 (15)	0.30477 (11)	0.0422 (3)	
C6	0.7641 (2)	1.3110 (2)	0.28366 (17)	0.0670 (5)	
H6	0.7739	1.3393	0.3429	0.080*	
C7	0.7427 (2)	1.41584 (19)	0.17152 (19)	0.0769 (6)	
H7	0.7374	1.5172	0.1548	0.092*	
C8	0.7291 (2)	1.37485 (16)	0.08406 (16)	0.0648 (4)	
H8	0.7136	1.4490	0.0091	0.078*	
C9	0.73808 (15)	1.22285 (14)	0.10554 (11)	0.0443 (3)	
H9	0.7292	1.1947	0.0461	0.053*	
C10	0.61443 (11)	0.95525 (12)	0.21822 (9)	0.0294 (2)	
H10	0.5964	0.8689	0.2757	0.035*	
C11	0.66671 (12)	0.90746 (15)	0.11868 (10)	0.0370 (2)	
H11A	0.6483	0.8173	0.1245	0.044*	
H11B	0.6070	1.0006	0.0465	0.044*	
C12	0.90212 (11)	0.81284 (11)	0.24386 (8)	0.02558 (18)	
C13	0.93053 (14)	0.73661 (15)	0.08136 (10)	0.0399 (3)	
H13A	1.0405	0.7158	0.0853	0.060*	
H13B	0.8829	0.7789	0.0033	0.060*	
H13C	0.9282	0.6357	0.1250	0.060*	
C14	0.46237 (12)	1.11772 (13)	0.18539 (10)	0.0347 (2)	
C15	0.2897 (3)	1.3079 (2)	0.25589 (17)	0.0936 (7)	
H15A	0.3143	1.3946	0.2131	0.140*	
H15B	0.2644	1.3125	0.3276	0.140*	
H15C	0.1986	1.3214	0.2135	0.140*	
C16	0.92790 (12)	0.63976 (11)	0.32962 (8)	0.0289 (2)	
C17	1.16767 (12)	0.63888 (13)	0.34810 (9)	0.0326 (2)	
C18	1.06891 (11)	0.79224 (12)	0.26043 (9)	0.0299 (2)	
C19	1.13393 (14)	0.89045 (15)	0.19952 (12)	0.0440 (3)	
H19	1.0693	0.9923	0.1397	0.053*	
C20	1.29723 (16)	0.83568 (18)	0.22851 (14)	0.0543 (3)	
H20	1.3422	0.9014	0.1880	0.065*	
C21	1.39322 (15)	0.68469 (19)	0.31685 (14)	0.0547 (4)	
H21	1.5024	0.6499	0.3353	0.066*	
C22	1.32993 (14)	0.58367 (17)	0.37874 (12)	0.0482 (3)	
H22	1.3946	0.4822	0.4389	0.058*	
N1	0.78263 (14)	1.03949 (13)	0.41018 (9)	0.0459 (3)	
N2	0.83966 (10)	0.85688 (10)	0.12758 (7)	0.02941 (18)	
N3	1.08062 (11)	0.55400 (11)	0.38933 (8)	0.0349 (2)	
H3	1.1191	0.4587	0.4458	0.042*	
O1	0.77037 (12)	0.79974 (10)	0.47206 (7)	0.0499 (2)	
O2	0.83142 (10)	0.58935 (10)	0.33842 (7)	0.0409 (2)	
O3	0.38293 (12)	1.20557 (12)	0.09240 (8)	0.0593 (3)	
O4	0.42723 (12)	1.15085 (11)	0.27609 (8)	0.0563 (3)	

### Atomic displacement parameters (Å^2^)

**Table:**

	*U*^11^	*U*^22^	*U*^33^	*U*^12^	*U*^13^	*U*^23^
C1	0.1180 (16)	0.0905 (13)	0.0559 (10)	−0.0537 (12)	0.0155 (10)	−0.0508 (10)
C2	0.0382 (5)	0.0283 (5)	0.0297 (5)	−0.0120 (4)	0.0025 (4)	−0.0110 (4)
C3	0.0304 (4)	0.0215 (4)	0.0268 (5)	−0.0134 (3)	0.0023 (3)	−0.0077 (3)
C4	0.0331 (5)	0.0223 (4)	0.0400 (6)	−0.0148 (4)	0.0053 (4)	−0.0098 (4)
C5	0.0475 (6)	0.0353 (6)	0.0548 (8)	−0.0246 (5)	0.0098 (5)	−0.0234 (5)
C6	0.0847 (11)	0.0541 (8)	0.0965 (13)	−0.0476 (8)	0.0291 (9)	−0.0476 (9)
C7	0.0947 (13)	0.0401 (7)	0.1167 (16)	−0.0464 (9)	0.0458 (11)	−0.0374 (9)
C8	0.0760 (10)	0.0301 (6)	0.0769 (11)	−0.0284 (7)	0.0299 (8)	−0.0089 (7)
C9	0.0500 (6)	0.0282 (5)	0.0453 (7)	−0.0189 (5)	0.0117 (5)	−0.0064 (5)
C10	0.0274 (4)	0.0264 (4)	0.0334 (5)	−0.0130 (4)	0.0027 (4)	−0.0112 (4)
C11	0.0291 (4)	0.0416 (6)	0.0421 (6)	−0.0132 (4)	−0.0001 (4)	−0.0234 (5)
C12	0.0269 (4)	0.0216 (4)	0.0264 (5)	−0.0127 (3)	0.0022 (3)	−0.0067 (3)
C13	0.0400 (5)	0.0421 (6)	0.0370 (6)	−0.0152 (5)	0.0088 (4)	−0.0216 (5)
C14	0.0307 (5)	0.0321 (5)	0.0385 (6)	−0.0134 (4)	0.0060 (4)	−0.0142 (4)
C15	0.0981 (14)	0.0496 (9)	0.0712 (11)	0.0100 (9)	0.0338 (10)	−0.0235 (8)
C16	0.0345 (5)	0.0230 (4)	0.0280 (5)	−0.0140 (4)	0.0065 (4)	−0.0093 (4)
C17	0.0308 (4)	0.0308 (5)	0.0313 (5)	−0.0122 (4)	0.0003 (4)	−0.0110 (4)
C18	0.0280 (4)	0.0275 (4)	0.0323 (5)	−0.0141 (4)	0.0019 (4)	−0.0093 (4)
C19	0.0375 (5)	0.0375 (6)	0.0529 (7)	−0.0237 (5)	0.0029 (5)	−0.0075 (5)
C20	0.0395 (6)	0.0593 (8)	0.0714 (10)	−0.0340 (6)	0.0086 (6)	−0.0215 (7)
C21	0.0313 (5)	0.0623 (8)	0.0714 (9)	−0.0229 (6)	0.0003 (6)	−0.0275 (7)
C22	0.0324 (5)	0.0454 (7)	0.0511 (7)	−0.0102 (5)	−0.0081 (5)	−0.0143 (6)
N1	0.0614 (6)	0.0455 (6)	0.0404 (6)	−0.0272 (5)	0.0053 (5)	−0.0250 (5)
N2	0.0285 (4)	0.0307 (4)	0.0262 (4)	−0.0124 (3)	0.0022 (3)	−0.0112 (3)
N3	0.0359 (4)	0.0236 (4)	0.0309 (5)	−0.0102 (3)	0.0015 (3)	−0.0025 (3)
O1	0.0732 (6)	0.0317 (4)	0.0308 (4)	−0.0189 (4)	0.0122 (4)	−0.0076 (3)
O2	0.0455 (4)	0.0325 (4)	0.0490 (5)	−0.0256 (3)	0.0095 (4)	−0.0125 (4)
O3	0.0468 (5)	0.0492 (5)	0.0499 (6)	0.0017 (4)	−0.0116 (4)	−0.0191 (5)
O4	0.0584 (6)	0.0415 (5)	0.0428 (5)	−0.0039 (4)	0.0154 (4)	−0.0178 (4)

### Geometric parameters (Å, º)

**Table:**

C1—N1	1.4514 (18)	C12—N2	1.4484 (13)
C1—H1A	0.9600	C12—C18	1.5028 (13)
C1—H1B	0.9600	C12—C16	1.5645 (13)
C1—H1C	0.9600	C13—N2	1.4548 (14)
C2—O1	1.2149 (13)	C13—H13A	0.9600
C2—N1	1.3529 (15)	C13—H13B	0.9600
C2—C3	1.5348 (14)	C13—H13C	0.9600
C3—C4	1.5052 (13)	C14—O3	1.1904 (14)
C3—C10	1.5497 (13)	C14—O4	1.3263 (14)
C3—C12	1.5558 (13)	C15—O4	1.4389 (17)
C4—C9	1.3775 (16)	C15—H15A	0.9600
C4—C5	1.3879 (17)	C15—H15B	0.9600
C5—C6	1.3808 (18)	C15—H15C	0.9600
C5—N1	1.4035 (17)	C16—O2	1.2112 (12)
C6—C7	1.376 (3)	C16—N3	1.3565 (13)
C6—H6	0.9300	C17—C22	1.3803 (15)
C7—C8	1.366 (3)	C17—C18	1.3899 (14)
C7—H7	0.9300	C17—N3	1.3946 (14)
C8—C9	1.4003 (18)	C18—C19	1.3748 (15)
C8—H8	0.9300	C19—C20	1.3871 (16)
C9—H9	0.9300	C19—H19	0.9300
C10—C14	1.5048 (14)	C20—C21	1.377 (2)
C10—C11	1.5276 (15)	C20—H20	0.9300
C10—H10	0.9800	C21—C22	1.386 (2)
C11—N2	1.4656 (13)	C21—H21	0.9300
C11—H11A	0.9700	C22—H22	0.9300
C11—H11B	0.9700	N3—H3	0.8600
			
N1—C1—H1A	109.5	C18—C12—C16	101.43 (7)
N1—C1—H1B	109.5	C3—C12—C16	110.59 (7)
H1A—C1—H1B	109.5	N2—C13—H13A	109.5
N1—C1—H1C	109.5	N2—C13—H13B	109.5
H1A—C1—H1C	109.5	H13A—C13—H13B	109.5
H1B—C1—H1C	109.5	N2—C13—H13C	109.5
O1—C2—N1	125.04 (11)	H13A—C13—H13C	109.5
O1—C2—C3	126.49 (10)	H13B—C13—H13C	109.5
N1—C2—C3	108.47 (9)	O3—C14—O4	123.86 (10)
C4—C3—C2	101.98 (8)	O3—C14—C10	126.06 (10)
C4—C3—C10	113.45 (8)	O4—C14—C10	110.09 (9)
C2—C3—C10	113.21 (8)	O4—C15—H15A	109.5
C4—C3—C12	114.98 (8)	O4—C15—H15B	109.5
C2—C3—C12	113.55 (8)	H15A—C15—H15B	109.5
C10—C3—C12	100.24 (7)	O4—C15—H15C	109.5
C9—C4—C5	120.03 (10)	H15A—C15—H15C	109.5
C9—C4—C3	131.23 (10)	H15B—C15—H15C	109.5
C5—C4—C3	108.51 (10)	O2—C16—N3	126.22 (9)
C6—C5—C4	122.02 (14)	O2—C16—C12	126.37 (9)
C6—C5—N1	128.08 (13)	N3—C16—C12	107.37 (8)
C4—C5—N1	109.88 (10)	C22—C17—C18	121.67 (11)
C7—C6—C5	117.35 (15)	C22—C17—N3	128.28 (10)
C7—C6—H6	121.3	C18—C17—N3	109.88 (9)
C5—C6—H6	121.3	C19—C18—C17	119.80 (10)
C8—C7—C6	121.67 (13)	C19—C18—C12	130.94 (9)
C8—C7—H7	119.2	C17—C18—C12	109.02 (8)
C6—C7—H7	119.2	C18—C19—C20	119.12 (11)
C7—C8—C9	120.99 (15)	C18—C19—H19	120.4
C7—C8—H8	119.5	C20—C19—H19	120.4
C9—C8—H8	119.5	C21—C20—C19	120.50 (12)
C4—C9—C8	117.92 (14)	C21—C20—H20	119.8
C4—C9—H9	121.0	C19—C20—H20	119.8
C8—C9—H9	121.0	C20—C21—C22	121.16 (11)
C14—C10—C11	114.04 (9)	C20—C21—H21	119.4
C14—C10—C3	113.06 (8)	C22—C21—H21	119.4
C11—C10—C3	104.49 (8)	C17—C22—C21	117.72 (12)
C14—C10—H10	108.3	C17—C22—H22	121.1
C11—C10—H10	108.3	C21—C22—H22	121.1
C3—C10—H10	108.3	C2—N1—C5	111.02 (10)
N2—C11—C10	105.43 (8)	C2—N1—C1	122.76 (12)
N2—C11—H11A	110.7	C5—N1—C1	125.37 (12)
C10—C11—H11A	110.7	C12—N2—C13	115.72 (8)
N2—C11—H11B	110.7	C12—N2—C11	108.13 (8)
C10—C11—H11B	110.7	C13—N2—C11	114.45 (8)
H11A—C11—H11B	108.8	C16—N3—C17	111.89 (8)
N2—C12—C18	114.11 (8)	C16—N3—H3	124.1
N2—C12—C3	100.86 (7)	C17—N3—H3	124.1
C18—C12—C3	117.03 (8)	C14—O4—C15	116.15 (12)
N2—C12—C16	113.33 (8)		
			
O1—C2—C3—C4	176.72 (11)	C18—C12—C16—O2	−171.33 (10)
N1—C2—C3—C4	−3.10 (11)	C3—C12—C16—O2	63.84 (13)
O1—C2—C3—C10	54.46 (14)	N2—C12—C16—N3	129.09 (9)
N1—C2—C3—C10	−125.36 (10)	C18—C12—C16—N3	6.33 (10)
O1—C2—C3—C12	−58.99 (14)	C3—C12—C16—N3	−118.50 (9)
N1—C2—C3—C12	121.20 (10)	C22—C17—C18—C19	2.17 (18)
C2—C3—C4—C9	−173.11 (11)	N3—C17—C18—C19	−173.57 (10)
C10—C3—C4—C9	−51.01 (15)	C22—C17—C18—C12	177.20 (10)
C12—C3—C4—C9	63.56 (15)	N3—C17—C18—C12	1.46 (12)
C2—C3—C4—C5	1.16 (11)	N2—C12—C18—C19	47.46 (15)
C10—C3—C4—C5	123.25 (10)	C3—C12—C18—C19	−69.93 (15)
C12—C3—C4—C5	−122.17 (10)	C16—C12—C18—C19	169.68 (12)
C9—C4—C5—C6	−2.22 (19)	N2—C12—C18—C17	−126.83 (9)
C3—C4—C5—C6	−177.24 (12)	C3—C12—C18—C17	115.78 (10)
C9—C4—C5—N1	176.11 (10)	C16—C12—C18—C17	−4.61 (11)
C3—C4—C5—N1	1.09 (13)	C17—C18—C19—C20	−1.35 (19)
C4—C5—C6—C7	1.7 (2)	C12—C18—C19—C20	−175.14 (12)
N1—C5—C6—C7	−176.26 (14)	C18—C19—C20—C21	0.2 (2)
C5—C6—C7—C8	−0.3 (3)	C19—C20—C21—C22	0.1 (2)
C6—C7—C8—C9	−0.6 (3)	C18—C17—C22—C21	−1.76 (19)
C5—C4—C9—C8	1.18 (18)	N3—C17—C22—C21	173.13 (12)
C3—C4—C9—C8	174.90 (12)	C20—C21—C22—C17	0.6 (2)
C7—C8—C9—C4	0.2 (2)	O1—C2—N1—C5	−175.84 (11)
C4—C3—C10—C14	−33.41 (12)	C3—C2—N1—C5	3.98 (13)
C2—C3—C10—C14	82.20 (10)	O1—C2—N1—C1	−5.9 (2)
C12—C3—C10—C14	−156.52 (8)	C3—C2—N1—C1	173.94 (13)
C4—C3—C10—C11	91.15 (10)	C6—C5—N1—C2	174.92 (14)
C2—C3—C10—C11	−153.24 (8)	C4—C5—N1—C2	−3.28 (15)
C12—C3—C10—C11	−31.96 (9)	C6—C5—N1—C1	5.3 (2)
C14—C10—C11—N2	132.27 (9)	C4—C5—N1—C1	−172.92 (14)
C3—C10—C11—N2	8.34 (11)	C18—C12—N2—C13	62.19 (11)
C4—C3—C12—N2	−77.70 (9)	C3—C12—N2—C13	−171.46 (8)
C2—C3—C12—N2	165.37 (8)	C16—C12—N2—C13	−53.25 (11)
C10—C3—C12—N2	44.33 (8)	C18—C12—N2—C11	−167.94 (8)
C4—C3—C12—C18	46.69 (12)	C3—C12—N2—C11	−41.58 (9)
C2—C3—C12—C18	−70.23 (11)	C16—C12—N2—C11	76.63 (10)
C10—C3—C12—C18	168.72 (8)	C10—C11—N2—C12	21.22 (11)
C4—C3—C12—C16	162.12 (8)	C10—C11—N2—C13	151.79 (9)
C2—C3—C12—C16	45.19 (10)	O2—C16—N3—C17	171.68 (10)
C10—C3—C12—C16	−75.85 (9)	C12—C16—N3—C17	−5.99 (11)
C11—C10—C14—O3	1.20 (16)	C22—C17—N3—C16	−172.33 (12)
C3—C10—C14—O3	120.39 (13)	C18—C17—N3—C16	3.04 (12)
C11—C10—C14—O4	−178.99 (9)	O3—C14—O4—C15	−3.9 (2)
C3—C10—C14—O4	−59.81 (12)	C10—C14—O4—C15	176.33 (15)
N2—C12—C16—O2	−48.57 (13)		

### Hydrogen-bond geometry (Å, º)

**Table:**

*D*—H···*A*	*D*—H	H···*A*	*D*···*A*	*D*—H···*A*
N3—H3···O1^i^	0.86	2.10	2.9107 (12)	157
C15—H15*B*···O1^ii^	0.96	2.47	3.363 (2)	155

Symmetry codes: (i) −*x*+2, −*y*+1, −*z*+1; (ii) −*x*+1, −*y*+2, −*z*+1.
